# Spontaneously Ruptured Idiopathic Mesenteric Hematoma: A Case Report With Review of Literature

**DOI:** 10.7759/cureus.29911

**Published:** 2022-10-04

**Authors:** Mohammed Alyaseen, Ali Toffaha, Ahmad L F Yasin, Hamza El Baba, Aryan Ahmed

**Affiliations:** 1 General Surgery, Hamad Medical Corporation, Doha, QAT; 2 Surgery, Hamad Medical Corporation, Doha, QAT; 3 Radiology, Hamad Medical Corporation, Doha, QAT

**Keywords:** exploratory laparotomy., hypotension, hemoperitoneum, ruptured mesenteric hematoma, idiopathic

## Abstract

Ruptured mesenteric hematoma is a rare entity that is not frequently reported in the literature. Spontaneous rupture of mesenteric hematoma has been vaguely described in a handful of cases. The majority of cases (unlike ours) had a reported etiology, like trauma, postoperative complications, connective tissue disease, and coagulopathy. To our knowledge, only two cases were reported for spontaneous rupture of idiopathic mesenteric hematoma. We, herein, present a 45-year-old male presented with abdominal pain, nausea, and vomiting associated with hypotension. His investigations, including abdominal computed tomography (CT) scan, showed a large lobulated mesenteric structure involving the small bowel mesentery, representing a mesenteric hematoma. Due to suspicion of intra-abdominal bleeding; we proceeded for exploratory laparotomy. Intraoperative findings showed a large mesenteric hematoma. The patient was stabilized after the evacuation of hematoma and no specific source of bleeding was identified. Ruptured idiopathic mesenteric hematoma is an extremely rare condition that poses a diagnostic challenge, it should be kept among deferential diagnoses, especially, in unstable patients with abdominal symptoms.

## Introduction

Spontaneous mesenteric hematoma is a rare entity with vague etiology [1]. It was first described in the early 1900s with proposed causes including trauma, connective tissue disease, coagulopathy, and arteriopathy [1]. Mesenteric hematoma is usually induced by traumatic events or postoperative complications. That being said, there have been multiple series in literature concluding that in 40% of the cases the bleeding vessels are not identified even after surgical exploration [2]. There is a delay in the management and treatment of such patients due to the vague presentation.

We report a case of spontaneously ruptured idiopathic mesenteric hematoma in a 45-year-old male that was managed surgically at Hamad General Hospital, Doha, Qatar. A literature review in a comprehensive approach was carried out. To our knowledge, this is the third reported case in literature for spontaneous rupture of idiopathic mesenteric hematoma.

## Case presentation

A 45-year-old male presented to the emergency department at Hamad General Hospital, Doha, Qatar, with abdominal pain for two days, associated with nausea, episodes of vomiting of food particles, and abdominal distension. He had no other gastrointestinal (GI) or genitourinary symptoms, no history of trauma or bleeding from other sites, and no history of anticoagulant or antiplatelets use. His past history was significant for type II diabetes mellitus (DM) and hypertension (HTN). The patient was a nonsmoker and nonalcoholic. Past surgical history was significant for left axillary abscess incision and drainage. On examination, the patient looked well and alert. His presenting vitals showed blood pressure (BP) of 91/58 mmHg, heart rate (HR) of 108 b/min, respiratory rate of 22, and temperature of 36.8 Celsius degree. On examining his abdomen, it was distended, with a palpable tender bulge in the left upper abdomen, and lower abdominal tenderness; however, there were no signs of diffuse peritonism. His labs showed white blood cell count of 12.5 x10^3^/µL (normal 4-10), hemoglobin (Hb) 6.5 g/dL (13-17), platelets count 126 x10^3^/µL (150-410), international normalized ratio (INR) 1.1, activated partial thromboplastin time (aPTT) 25.4 seconds (25-36), and lactic acid 3.5 mmol/L (<2.2).

**Figure 1:**
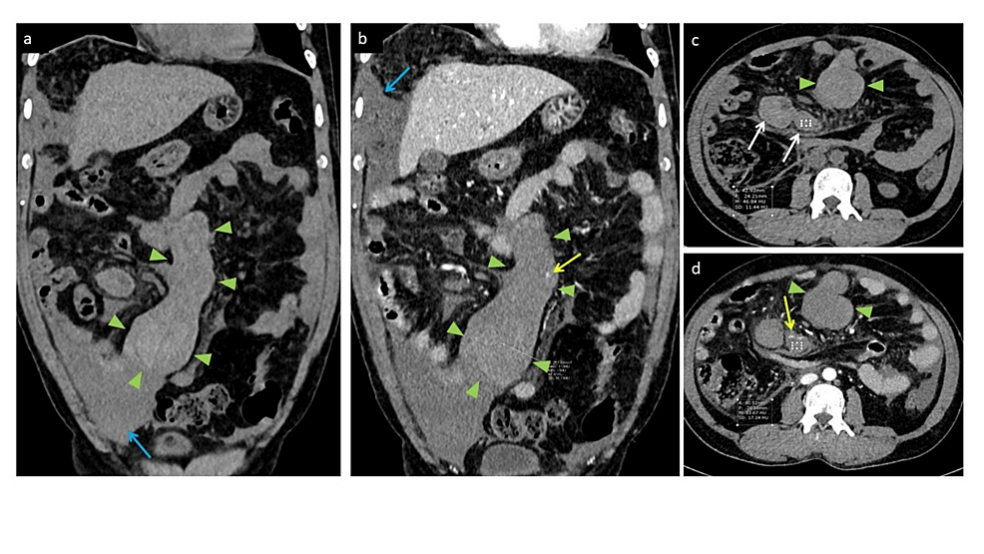
Non-contrasted (a, c) and contrasted (b, d) abdominal computed tomography A large lobulated mesenteric structure (arrowheads) involving the small bowel mesentery extending from the mesenteric root towards the right iliac fossa and measures 15 cm at maximal dimension. This structure shows high density in the non-contrast study and no post-contrast enhancement with surrounding fat stranding representing a mesenteric hematoma. This hematoma appears to displace the surrounding small bowels with no evidence of bowel wall thickening, decreased enhancement or intestinal obstruction. Other two smaller rounded adjacent similar hematomas (white arrows) are noted to the right side. Tiny contrast blushes were noted within these hematomas (yellow arrows) which suggest possible contrast extravasation. Mild amount of free fluid in the upper and lower abdomen (white arrows).

Abdomen and chest x-rays were normal. Bedside ultrasound showed the presence of free fluid in the pelvis. Computed tomography (CT) scan of the abdomen showed a 15-cm structure originating from the small bowel mesentery, extending from the mesenteric root towards the right iliac fossa, likely representing a mesenteric hematoma and moderate amount of fluids in the abdomen, possibly hemoperitoneum. Another differential diagnosis on the CT scan was a soft tissue mass that ruptured and caused this bleeding (Figures 1a-1d).

After initial resuscitation, the patient initially responded and had his CT scan. However, he started deteriorating again; hence, he was taken to the operating theater for exploratory laparotomy, intra-operative findings were hemoperitoneum (two liters of blood), mesenteric hematoma involving the mesentery of the small bowel extending to the root near superior mesenteric artery (SMA) origin, no actively bleeding vessel was identified. Evacuation of the hematoma was undertaken, and the abdomen was left with temporary closure (ABTHERA vac) for a second look later, no definite closure was undertaken as the patient was not stable and no clear bleeding source was identified or controlled.

The patient was shifted to the intensive care unit (ICU) postoperatively. Second look laparotomy showed healthy bowel, serosal hematoma involving the hepatic flexure and mesenteric hematoma with no active bleeding, approximation of the rectus sheath was not possible; hence, the decision is made for ABTHERA vac dressing again, further diuresis to resolve bowel edema in order to allow approximation of the fascia, so planned for re-look laparotomy after 48 hours.

The third look showed a dry site of previous hematoma evacuation, resolving hematoma of the mesocolon, no active bleeding and the bowel was healthy, closure of the abdomen with the posterior component separation technique was undertaken as it was not possible to bring the sheath in the midline despite all conservative measure including diuresis and muscle relaxation. On the same day, the patient developed hypotension (BP 74/50), tachycardia (HR 115 b/min), and a drop in Hb to 6 mg/dL (from 8.6 mg/dL), resuscitation was started, and he was shifted to the operating theater, where bleeding from the left inferior epigastric artery was identified, controlled and vac dressing was applied. Three days later, closure of the abdomen using anterior component separation was done. The patient then recovered gradually with the uneventful course, his wound was healing well, and he was discharged after 22 days of his initial presentation with stable vital signs and hemoglobin.

## Discussion

Mesenteric hematoma in itself is a rare cause of intraperitoneal hemorrhage due to bleeding from peripheral mesenteric vessels [3]. There are different etiologies for the development of such pathologies as abdominal trauma, pancreatitis, connective tissue disorders, post-surgical intervention, or a complication of anticoagulant medications [3]. Mesenteric hematoma is labeled as idiopathic in the absence of the previously mentioned causes [4].

Idiopathic mesenteric hematoma is an extremely rare pathology that may have vague presentations [5]. Majority of patients with mesenteric hematoma present with nonspecific symptoms like generalized abdominal pain, hypotension due to hemorrhage, and in some cases melena and/or hematemesis if the rupture is into the lumen of the small bowel [5]. The onset of the symptoms may vary as the bleeding can induce hypotension and/or hypovolemic shock [5]. Our patient presented with abdominal pain, and early features of hemorrhagic shock, which is in agreement with other reported cases in the literature (Table 1).

**Table 1:** Summary of characteristics of current case and other reported cases of ruptured spontaneous mesenteric hematoma identified from the review of the literature. *For space considerations, only the first author is cited, Abd: Abdomen; Const: Constipation; DM: Diabetes mellitus; F: Female; Hb: Hemoglobin; HTN: Hypertension; Intra-op: Intraoperative findings; LUQ: Left upper quadrant; M: Male; NR: Not reported; PE: Physical examination; PH: Past history; PPT: Presentation; SB: Small bowel; Tran: Transfusion; U: Units; US: ultrasound.

Study*	Sex (M/F)	Age	PH	PPT	PE	Labs	US	CT	Surgery	Intra-op	Histology	Follow-up
Current study Qatar (2021)	M	45	DM HTN	Abd pain Vomiting Abd distension	Distended Abd Tender palpable mass	Hb 6.5 g/dL	Free fluid in the pelvis	hyper dense mass 15 X 3.8 cm	Exploration laparotomy	Mesenteric hematoma Hemoperitoneum	NR	Uneventful
Ashrafian (2014) London [4]	F	44	Crohn’s disease. HTN	Abd pain and bloating with Const.	initial	Initial Hb 11.7 g/dL Repeat Hb 8.6 g/dL	NR	SB dilatation, 10 cm distal ileal stricture and a LUQ Abd mass 9 × 12 × 20cm	Laparotomy	Large hematoma within the mesentery of the mid-jejunum that corresponded to the mass demonstrated on CT	NR	Uneventful
Shikata (2016) Japan [2]	M	75	Duodenal ulcer Acute pancreatitis	Anal bleeding	NR	Initial Hb 8.2 g/dL Repeat Hb after 4 U tran. is 6.7 g/dL	NR	Low density mass (3 cm) suggesting a hematoma or an aneurysm.	Emergency laparotomy	Mesenteric hematoma	Capsular formation, fibrosis, hemosiderin deposits indicating chronic hematoma.	NR

CT abdomen with contrast is the diagnostic modality of choice to reveal mesenteric hematoma [5]. Imaging modalities can be utilized to rule out other causes of abdominal pain like abdominal aortic aneurysm, acute pancreatitis, and mesenteric ischemia [5].

Management of mesenteric hematoma is variable as it depends on the patient’s stability. If the patient is hemodynamically stable, he can be treated conservatively mainly with the absence of active bleeding [6]. In clinically stable patients with extravasation, CT angiography can be beneficial to visualize the source of bleeding as well as embolization of the bleeding vessels. As a result, this can save patients from major laparotomies. Regular follow-up with CT scans is required in a non-operative approach to follow the size of the hematoma and to correlate with the hemodynamics of the patient [7]. Laparoscopic exploration can be more beneficial than laparotomy in rather hemodynamically stable patients to decrease pulmonary related complications, wound infections and for better visualization of the abdominal cavity [6]. On the other hand, if the patient presents with hypovolemic shock and hypotension with no response to aggressive hydration; then surgical management with exploration laparotomy is indicated to assess and manage the source of bleeding [6].

Our patient presented with hypotension and features of hemodynamic instability. Due to the high suspicion of intra-abdominal catastrophe; bed side ultrasound demonstrated free fluid in the pelvis. Then an urgent CT scan was done after initial response to fluids. However, latter on his response to initial resuscitation was not sustained, the patient was explored via a midline laparotomy. Following abdominal closure; he was sent home in a good condition and a smooth postoperative course in six months follow up.

## Conclusions

Ruptured idiopathic mesenteric hematoma is an extremely rare clinical entity in patients with abdominal pain presenting to the emergency department. An abdominal CT scan with contrast is the diagnostic modality of choice to confirm the diagnosis as well as to rule out other potential causes of abdominal pain. Admission is warranted for all patients and the hemodynamic status of the patient guides the management. Bleeding can stop spontaneously or by therapeutic interventions. Thorough evaluation for the cause of bleeding is mandated in all cases to avoid recurrence.
